# Electrophysiological features: The next precise step for *SCN2A* developmental epileptic encephalopathy

**DOI:** 10.1002/mgg3.1250

**Published:** 2020-05-13

**Authors:** Pu Miao, Siyang Tang, Jia Ye, Jianda Wang, Yuting Lou, Bijun Zhang, Xiaoxiao Xu, Xiaoquan Chen, Yuezhou Li, Jianhua Feng

**Affiliations:** ^1^ Second Affiliated Hospital Zhejiang University School of Medicine Hangzhou China; ^2^ Children’s Hospital and Department of Biophysics, National Clinical Research Center for Child Health, NHC and CAMS Key Laboratory of Medical Neurobiology Zhejiang University School of Medicine Hangzhou China

**Keywords:** developmental epileptic encephalopathy, patch‐clamp, precise treatment, *SCN2A*

## Abstract

**Background:**

To investigate the relationships among phenotypes, genotypes, and funotypes of *SCN2A*‐related developmental epileptic encephalopathy (DEE).

**Methods:**

We enrolled five DEE patients with five de novo variants of the *SCN2A*. Functional analysis and pharmacological features of Nav1.2 channel protein expressed in HEK293T cells were characterized by whole‐cell patch‐clamp recording.

**Results:**

The phenotypes of c.4712T>C(p. I1571T), c.2995G>A(p.E999K), and c.4015A>G(p. N1339D) variants showed similar characteristics, including early seizure onset with severe to profound intellectual disability. Electrophysiological recordings revealed a hyperpolarizing shift in the voltage dependence of the activation curve and smaller recovery time constants of fast‐inactivation than in wild type, indicating a prominent gain of function (GOF). Moreover, pharmacological electrophysiology showed that phenytoin inhibited over a 70% peak current and was more effective than oxcarbazepine and carbamazepine. In contrast, c.4972C>T (p.P1658S) and c.5317G>A (p.A1773T) led to loss of function (LOF) changes, showing reduced current density and enhanced fast inactivation. Both showed seizure onset after 3 months of age with moderate development delay. Interestingly, we discovered that choreoathetosis was a specific phenotype feature.

**Conclusion:**

These findings provided the insights into the phenotype–genotype–funotype relationships of *SCN2A*‐related DEE. The preliminary evaluation using the distinct hints of GOF and LOF helped plan the treatment, and the next precise step should be electrophysiological study.

## Graphical abstract

In this manuscript, we report the clinical and electrophysiological results of five patients of *SCN2A*‐related developmental epileptic encephalopathy (DEE). Furthermore, we reviewed published data and combined our results to propose a protocol of diagnosis and treatment for *SCN2A*‐related DEE. These findings provided the insights into the phenotype‐genotype relationships and the electrophysiological function‐based precision treatment of *SCN2A*‐related DEE.

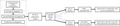

## 1 INTRODUCTION

Pathogenic variants of the *SCN2A*（OMIM#182390） are highly associated with a broad phenotypic spectrum. This ranges from developmental epileptic encephalopathy (DEE), including Ohtahara syndrome, infantile spasms, epilepsy of infancy with migrating focal seizures, and Lennox–Gastaut syndrome, to benign (familial) infantile seizures (B(F)NIS) and autistic spectrum disorder (ASD)/intellectual disability (ID) (Begemann et al., 2019; Ben‐Shalom et al., 2017; Wolff et al., 2017). As the largest group of *SCN2A* carriers, *SCN2A*‐related infantile DEE presents as age‐dependent refractory epilepsy and has a poor prognosis with severe developmental delay. The frequency of *SCN2A*‐related DEE in the general population and literature is approximately 1.4 per 100,000 births and thus represents a heavy social and family burden (Sanders et al., 2018).

The *SCN2A* encodes the α‐subunit of human voltage‐gated sodium channel Nav1.2, which is mainly expressed throughout the human central nervous system. In the early development period, Nav1.2 is the only sodium channel isoform expressed in the axon initial segment, that supposedly plays an important role in promoting propagation of action potential to soma and dendrites (Gazina et al., 2015; Laezza et al., 2009; Rush, Dib‐Hajj, & Waxman, 2005). For *SCN2A*, more than 700 variants have been identified (https://www.ncbi.nlm.nih.gov/clinvar); however, only a small number have been studied functionally. The gain of function (GOF) was detected in variants from either *SCN2A*‐related DEE (Kamiya et al., 2004; Ogiwara et al., 2009; Wolff et al., 2017) or B(F)NIS phenotype (Lauxmann et al., 2013; Schwarz et al., 2016). The reasonable explanation was that the pathogenic variants found in B(F)NIS increased the neuronal excitability during early development but not in the mature brain. The variants observed in DEE could result in a greater degree of neuronal excitability that persists in the mature brain. In contrast, opposing effects of loss of function (LOF) on Nav1.2 function leading to a reduction in neuronal excitability in pyramidal neurons have been observed in the majority of missense variants of ASD (Ben‐Shalom et al., 2017). In fact, pathogenic variants, such as R853Q (Berecki et al., 2018) or G899S (Wolff et al., 2017), identified in *SCN2A*‐related DEE patients were previously affirmed previously to cause LOF changes. The neuropathological mechanisms explaining why both GOF and LOF could lead to DEE phenotype remains largely unknown. However, the distinct difference of phenotype between GOF and LOF should be investigated to guide precise treatments, because patients with LOF variant should be avoid sodium‐channel blockers (SCBs) (Oyrer et al., 2018).

Here, we described the phenotypes, genotypes, and funotypes of a cohort of five DEE patients with three new variants of the *SCN2A* namely, c.4712T>C (p.I1571T); c.4972C>T(p.P1658S); c.5317G>A(p.A1773T); and two hot spots c.2995G>A(p.E999K) and c.4015A>G(p.N1339D). We then reviewed the published functionally studied variants investigated in *SCN2A*‐realated DEE patients to help us conclude the distinct differences in phenotypes between GOF and LOF variants.

## 2 MATERIALS AND METHODS

### 2.1 Ethical compliance

All procedures were approved by the Ethics Committee of the Second Affiliated Hospital of School of Medicine, Zhejiang University, China.

### 2.2 Patients and variants

In this study, five patients from a cohort of childhood epilepsy without nongenetic factors, such as an acquired brain injury (including traumatic brain injury, encephalitis, vasculitis, hypoxia, abscess, neoplasm, metabolic disturbance, and toxicity) were included. Patient 3 and patient 4 underwent a targeted panel sequencing (Miao et al., 2018), while the others underwent whole‐exome sequencing (WES). Sanger sequencing was employed to validate these variants among the patient and parents. The pathogenicities of these variants of the *SCN2A* were assessed according to the standards recommended by the American College of Medical Genetic and Genomics.

### 2.3 Plasmid constructs

The cDNA of human Nav1.2α was purchased from Addgene and human Nav β1 and β2 subunits was generously provided by Dr. S. C. Cannon and Dr. S. G. Waxman. WT Nav1.2 was subcloned into a pCMV vector. Variants were introduced into pCMV‐Nav1.2 using a ClonExpress II One Step Cloning Kit (vazyme). The Nav β1 and β2 subunits were subcloned into pIRES2‐EGFP and pIRES2‐mCherry vectors, respectively. The ORF of all plasmids were confirmed by sequencing full length before transfection.

### 2.4 Cell culture and transfection

HEK293T cells were obtained from ATCC and maintained at 37°C with 5% CO_2_ in Dulbecco's modified Eagle's medium supplemented with 10% fetal bovine serum (Gibco). The expression of hNav1.2 and the accessory β1 and β2 subunits was achieved by using transient transfection with Lipofectamine 2000 (Invitrogen). Electrophysiological recordings of both green and red fluorescent cells were made 24 hr after transfection.

### 2.5 Electrophysiology

Whole‐cell voltage‐clamp experiments were used to examine the voltage‐gated Na^+^ currents (Sakmann & Neher, 1984). All voltage‐clamp experiments were performed at room temperature. The data were collected using an Axon multiclamp 700B, Digidata1440A (Axon Instruments). Patch pipettes were pulled and fire polished to a pipette resistance of 1.1‒2.0MΩ. The pipette solution contained 10 mM NaF, 110 mM CsF, 20 mM CsCl, 2 mM EGTA, and 10 mM HEPES with pH adjusted to 7.35 with CsOH and osmolarity adjusted to 310 mOsmol/kg with sucrose. The bath solution contained 145 mM NaCl, 4 mM KCl, 1.8 mM CaCl_2_, 1 mM MgCl_2_, 10 mM d‐(+)glucose, and 10 mM HEPES with pH adjusted to 7.35 with NaOH and osmolarity adjusted to 310 mOsmol/kg with sucrose. For the pharmacologic experiments, all the reagents were obtained from Sigma‐Aldrich and dissolved using dimethyl sulfoxide to 100mM as a stock solution. The reagents were diluted from stock solution using bath solution prior to the electrophysiology recording. The extracellular solution contained the same final concentration of dimethyl sulfoxide (0.1%). To minimize voltage errors, we focused on data from cells expressing maximal peak Na^+^ currents amplitudes between 1 and 8 nA. By using low resistance pipettes and 90‒95% series resistance compensation (Herzog, Cummins, Ghassemi, Dib‐Hajj, & Waxman, 2003), the average series resistance in these cells was 1.9 ± 0.3 MΩ and the estimated maximum voltage error of the recordings was 1.8 ± 0.5 mV.

### 2.6 Statistics method

All values were expressed as means ± standard error of mean, and the one way ANOVA and the Dunnett's post hoc test was used for statistical analysis.

### 2.7 Literature review

We selected patients diagnosed with DEE, with confirmed pathogenic *SCN2A* variants and electrophysiological functional data by performing a search using the term “*SCN2A*” on PubMed (up to July 30, 2019).

## 3 RESULTS

### 3.1 Phenotype characteristics of patients and variant locations

All five patients were born at term and had no reported hypoxic‐ischemic encephalopathy or asphyxia in the neonatal period. Early infantile epilepsy onset (<3 months of age) was observed in three patients, and the other two patients had seizures with later onset (≥3 months of age).

#### 3.1.1 Patient 1

Patient 1 was a 2‐year‐ and 3‐month‐old boy who presented with tonic seizure onset 2 days after birth followed by spasms at 1‐month of age. An electroencephalogram (EEG) showed burst suppression followed by numerous clusters of infantile spasms and a hypsarrhythmia pattern. Magnetic resonance imaging (MRI) results were normal. He had severe developmental delay. He was initially treated with phenobarbital (PB) without effect. Subsequent treatments included topiramate (TPM), valproate (VPA), and levetiracetam (LEV). For ongoing spasms, vigabatrin (VGB) was administered and produced a decrease in seizures. He was diagnosed with *SCN2A* encephalopathy with the c.4712T>C (NM_001040142, p. I1571T) variant, and oxcarbazepine (OXC) was then gradually administered, resulting in seizure‐free status and initial improvement in intellectual and motor development.

#### 3.1.2 Patient 2

Patient 2 was a 4‐year‐ and 9‐month‐old girl. At 3 months old, she presented with unilateral limb tonic seizure onset lasting for nearly 10 s to 2 min and accompanied by vomiting. EEG showed a sharp wave in the left occipital and temporal lobes. Cranial MRI presented normal results, while positron emission tomography‐computed tomography (PET‐CT) showed lower glucose metabolism in the left temporal lobe. She was initially treated with VPA. Later, OXC and LEV were subsequently added without effect. Then, given the lack of response of OXC and LEV, a ketogenic diet was added but had a poor response. By the time she had reached 3 years old, the unilateral limb tonic seizures had stopped. She presented the involuntary dancing motions with crying and unaware panic motions; these neurological features continued to be accompanied by vomiting lasting for nearly 2 days with dancing motions occurring approximately one time per 10 days. Vitamin B12 was added without effect. Genetic analysis found a rare de novo variant (NM_001040142, c.4972C > T, p.P1658S) which was predicted to be damaging by in silico analysis, and our functional investigation showed that this is a LOF variant. Given the results of our electrophysiological study, we stopped administering OXC. She was similarly weaned off LEV and a ketogenic diet. At the last follow‐up (4 years old), she had moderate developmental delay and was able to walk, express 3‒5 words on her mind and control urination. No autism manifestations were reported.

#### 3.1.3 Patient 3

Patient 3 was a 1‐year‐ and 10‐month‐old boy. He had seizure onset at 1 day after birth that presented as clusters of infantile spasms with limb extension occurring at a rate of approximately 10 clusters per day (2‒3 spasms per cluster). Tonic‐spasm onset occurred at 1‐month‐old. EEG showed burst suppression and epilepsy of infancy with migrating focal seizures, and he had a normal cranial MRI. He was diagnosed with Ohtahara syndrome with serious developmental delay. He was first treated with PB, followed by LEV, TPM, and VGB, with poor outcomes. OXC achieved partial remission. EEG shifted to a hypsarrhythmia pattern. For ongoing spasms, his parents chose a ketogenic diet instead of adrenocorticotropic hormone (ACTH) or oral prednisone because of a persistent hepatitis B virus (HBV) infection. However, the seizures remained refractory to treatment, and genetic analysis identified a rare variant (NM_001040142, c.2995G>A, p.E999K). We started to administer another SCB, phenytoin (PHT). Ultimately, the seizures ceased after PHT was titrated to 12.5 mg kg^‐1^ day^‐1^, and EEG improved without a hypsarrhythmia pattern.

#### 3.1.4 Patient 4

Patient 4 was a 2‐year‐old girl. At 2 months after birth, her parents noted abnormal paroxysmal limb gestures that occurred multiple times per day with irritability. Tonic seizure onset became frequent, at nearly 30 times per day. EEG showed burst suppression with a normal cranial MRI. She failed to respond to multiple antiepileptic drugs (AEDs), such as LEV, OXC, VPA, TPM, and VGB. At 8 months, she presented at our hospital with tonic seizures and spasms that had increased to nearly hundreds of times per day. A second cranial MRI scan showed encephalatrophy in the frontal lobe. Genetic analysis identified a rare variant (NM_001040142, c.4015A>G, p.N1339D). We began to administer PHT, and the patient miraculously achieved seizure‐free status until PHT was titrated to 14 mg kg^‐1^ day^‐1^. However, she still has severe developmental delay and is unable to hold up her head.

#### 3.1.5 Patient 5

Patient 5 was a 4‐year‐ and 10‐month‐old girl. She had spasms onset at 11 months old with developmental regression, including loss of smiling, rolling, and sitting. EEG showed a slow background and multiple spikes and sharp waves in the generalized lobes. She was treated with VPA, TPM, VGA, OXC, and oral prednisone. OXC was later stopped following exacerbation of seizures. Genetic analysis revealed a de novo heterozygous missense pathogenic variant (NM_001040142, c.5317G>A, p. A1773T) in *SCN2A*. Her seizures had stopped by 2 years and 5 months old, and she was found to have involuntary dancing motions, similar to patient 2. At the last follow‐up (3 years old), the patient remained seizure‐free while on treatment with VPA, TPM, VGA, and LEV. She could walk by herself and say mama and dada unconsciously.

### 3.2 Details of these variants

**FIGURE 1:**
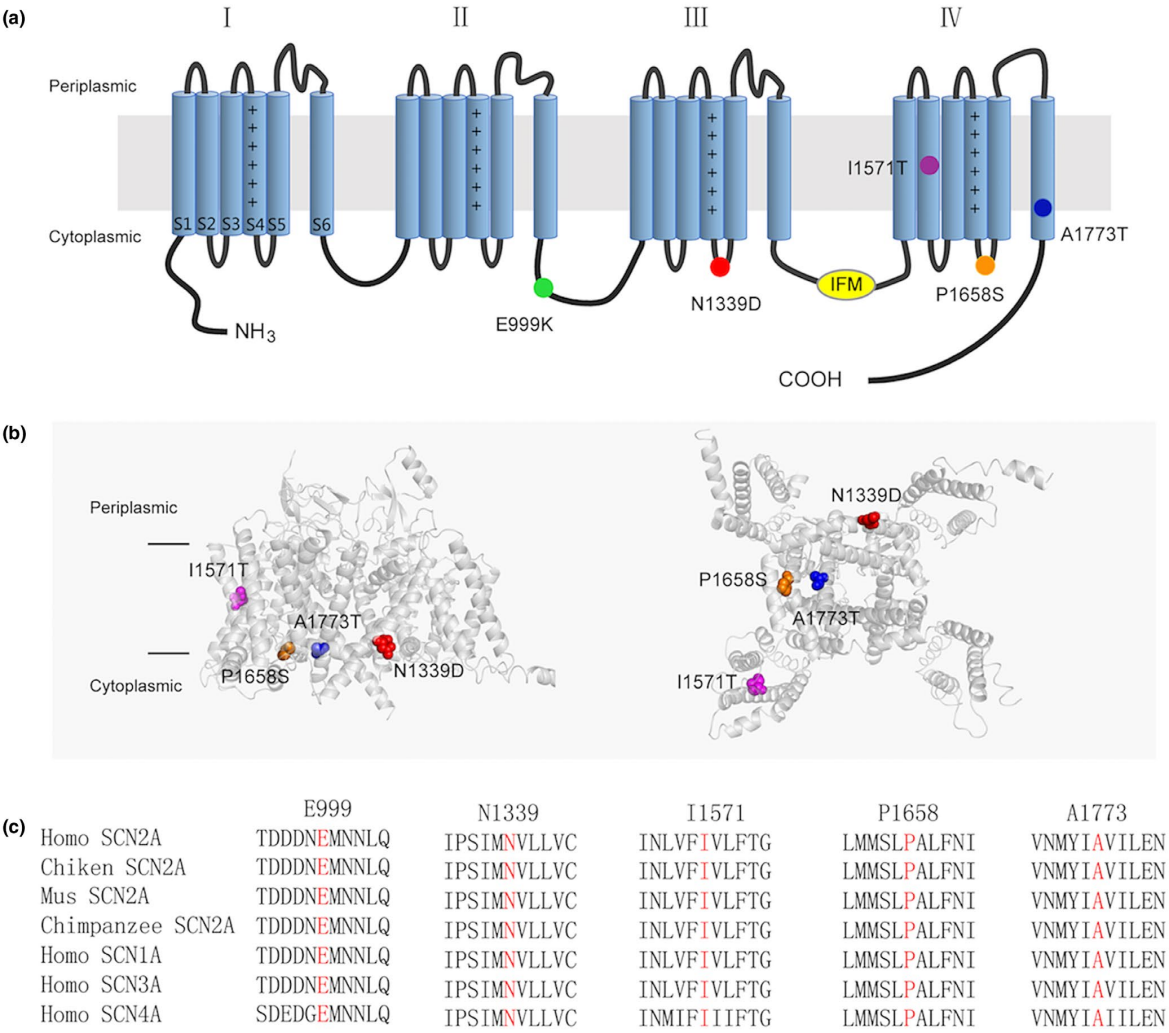
Locations of Epilepsy‐associated Nav1.2 mutations. (a) Topology diagram of the human Nav1.2 channel α subunit. The α subunit consists of four homologous domains (I–IV), each of which contains six transmembrane regions (S1–S6). Plus signs in S4 represent the positively charged voltage sensor (containing a number of arginines or lysines). Segments S1−S3 and S4 form the voltage‐sensing domain. S5–S6 in conjunction with their extracellular linker constitute the channel pore. The intracellular loop connecting III/S6 and IV/S1 contains the isoleucine, phenylalanine, and methionine (IFM) domain involved in channel inactivation (yellow). The locations of the mutated residues described in the present report are shown as circles in different colors. (b) Structure of the human Nav1.2 α subunit (PDB:6J8E). The left is the side view and the right is the bottom view. The mutations are shown as CPK style in different colors. The p.E999K mutation is located in the loop between II and III, which are not resolved in the original structure, so it cannot be exhibited. (c) Protein alignment showing that the affected residues (highlighted in red) are conserved cross in human Nav homologous and various species orthologs

The locations of the five variants within the topological and resolved Cry‐EM structure of Nav1.2 protein are shown in Figure 1a,b. An alignment of amino acid sequences showed that all five mutant sites were highly conserved across other human sodium channels and multiple species (Figure 1c) and had damaging in silico predictions of CADD and Polyphen. Moreover, they were all rare variants that were not found in the Gnomad database（Table S1）. Therefore, these variants were likely to change the function of the channel.

### 3.3 Functional study

**FIGURE 2:**
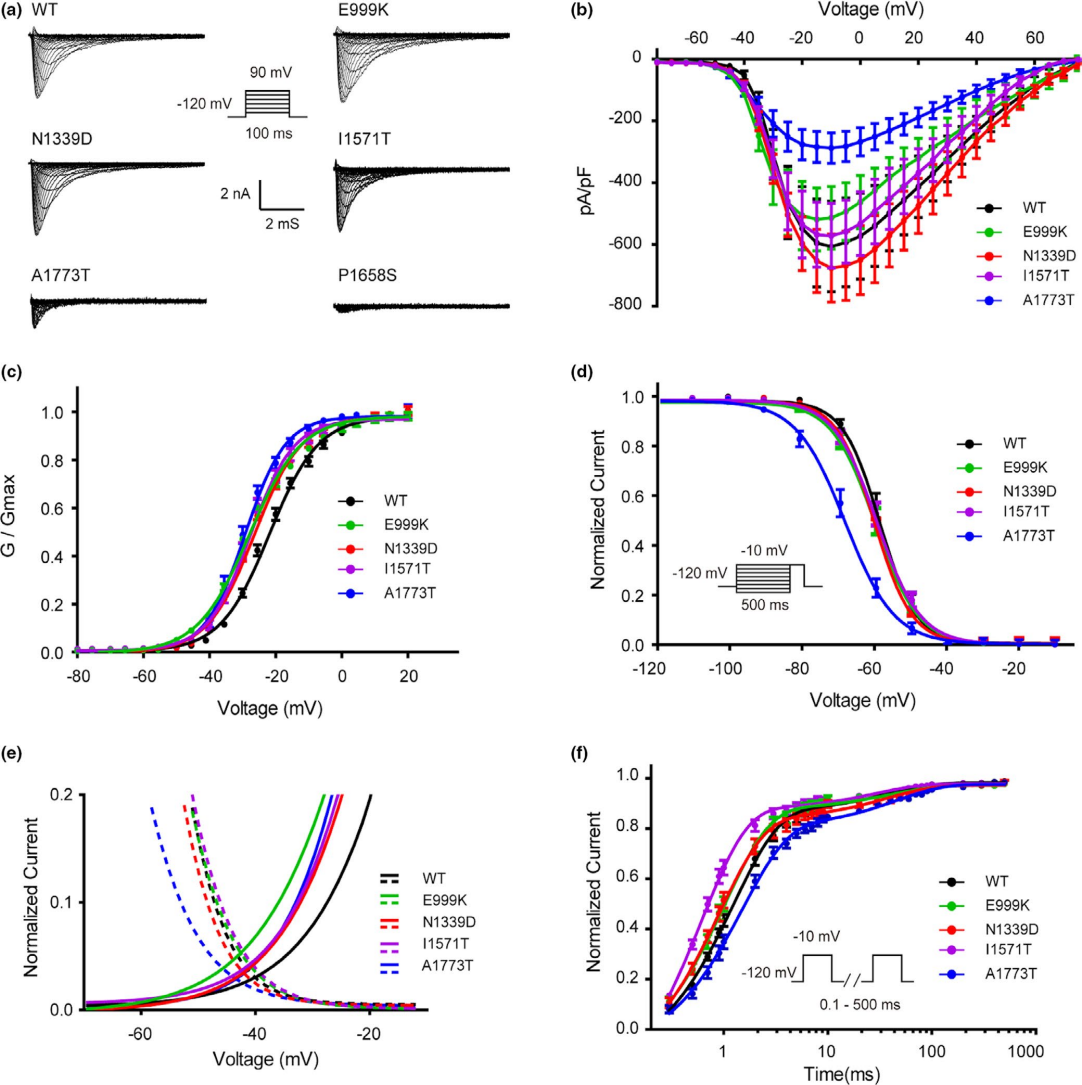
Epilepsy‐associated mutations alter the function of the Nav1.2 channel. (a) Representative whole‐cell patch‐clamp traces of voltage‐dependent currents recorded from HEK293T cells transfected with either Nav1.2 wild‐type or mutant channels. Cells were held at −120 mV and sodium currents were evoked by a series of depolarizing pulses (100 ms) to potentials ranging from −80 mV to +90 mV in steps of 5 mV (inset). (b) Normalized I‒V curves of peak sodium current density (in pA/pF) versus voltage for WT and mutations. (c) Voltage dependence of activation obtained by plotting the normalized conductance against test potentials with equation G/G_max_ = 1/(1 + exp(V_0.5_ − V)/k, where the G_max_ is the maximum conductance, V_0.5_ is the half‐maximal activation potential and k is the slope factor. the curve is fitted to a Boltzmann function. (d) Steady state of fast inactivation of WT and mutant Nav1.2 channels. The voltage dependence of fast inactivation was assessed by applying a double‐pulse protocol: 500‐ms prepulses were applied from −150 to 0 mV in steps of 10 mV and followed by a test pulse to −10 mV (inset). The steady‐state fast inactivation curve was fitted by the Boltzmann equation (I/Imax = {1 + exp[(V − V_0.5_)/k]} − 1). (e) Window currents of WT the mutations. Activation curves (fraction of maximum conductance, G/G_max_) and steady‐state inactivation (fraction of maximum current, I/Imax) of WT and the mutations are enlarged and plotted together to show the window currents. (f) The time course of recovery from fast inactivation. Recovery from fast inactivation was assessed by a two‐pulse recovery protocol with varying time intervals between a 500‐ms inactivating prepulse and a test pulse to −10 mV (inset). The time course of recovery from inactivation was fitted with a double‐exponential function to generate τ1 and τ2. All fitting results are listed in Table 1. Data are presented as the mean ± *SEM*

Among these patients, we identified five variants in the *SCN2A* including three de novo variants of I1571T, P1658S, and N1339D, and none of these variants have been functionally studied before. To determine the functional effects of these variants on the Nav1.2 channel, whole‐cell patch clamp recordings were performed and the results compared with those obtained for the wild‐type (WT) channel. Electrophysiology results showed that the E999K, N1339D, and I1571T variants produced a hyperpolarizing shift in the voltage dependence of the activation curve. Compared to the WT Nav1.2 channel, the half‐maximal activation potential (V_0.5_) shifted by 5~8 mV in a hyperpolarized direction in these variants (Figure 2a‐c). Moreover, the fast‐inactivation recovery time constant τ1 obtained for E999K, N1339D, and I1571T and the τ2 obtained for I1571T were smaller than those obtained in the WT group (Figure 2f, Table 1), indicating that these variants led to an accelerated recovery from fast inactivation. Furthermore, E999K showed increased the window currents (Figure 2e). These results indicated that E999K, N1339D, and I1571T variants produced GOF changes.

**TABLE 1:** Functional characteristics of Nav1.2 channel

	Peak amplitude of Na^+^ current	Voltage dependence of activation		Voltage dependence of fast inactivation		Recovery from fast inactivation	
	pA/pF	V_0.5_, mV	k, mV	V_0.5_, mV	k, mV	τ1, ms	τ2, ms
WT	−607.14 ± 135.84	−21.76 ± 1.46	7.40 ± 0.87	−58.57 ± 1.40	4.85 ± 0.30	1.25 ± 0.11	39.07 ± 6.62
E999K	−513.57 ± 101.72	−28.56 ± 1.25^**^	8.03 ± 0.52	−59.86 ± 1.62	5.22 ± 0.49	1.03 ± 0.10^*^	41.03 ± 6.02
N1339D	−676.55 ± 102.04	−26.43 ± 1.16^**^	7.10 ± 0.56	−60.30 ± 0.97	4.86 ± 0.59	0.95 ± 0.17^**^	37.52 ± 7.36
I1571T	−572.07 ± 104.75	−27.57 ± 1.82^**^	6.70 ± 0.72	−59.60 ± 1.30	4.93 ± 0.47	0.61 ± 0.15^**^	25.02 ± 6.14^**^
A1773T	−287.91 ± 49.32^*^*	−29.23 ± 2.04^**^	6.05 ± 0.75^*^	−68.76 ± 2.01^**^	5.4 ± 0.74	1.60 ± 0.28^**^	46.32 ± 5.40

Note

V_0.5_ is voltage of half‐maximal (in‐) activation; k is slope factor; τ is time constant. Data are presented as means ± *SEM*, *n* = 12–15. *p < .05 and **p < .01 versus WT were determined by a Dunnett's post hoc test after a one way ANOVA.

In contrast, the P1658S of Nav1.2 causes a complete LOF variant that did not produce detectable channel currents (Figure 2a). Compared to WT, the A1773T mutant channel exhibited a significant hyperpolarizing shift in the voltage dependence of the activation curve. However, this variant also showed a depolarizing shift of steady‐state fast inactivation and its current density was substantially reduced. The fast inactivation curve of A1773T channel exhibited ~10 mV hyperpolarizing shift of V_0.5_ (−68.76 ± 2.01 mV) as compared to WT (−58.57 ± 1.40 mV) (Figure 2d). Moreover, the recovery time constant τ1 from fast‐inactivation was significantly larger for the A1773T variant (1.60 ± 0.28 ms) as compared to the WT (1.25 ± 0.11 ms). Therefore, these data showed that the A1773T variant produced more LOF than GOF effects.

### 3.4 Molecular‐antiepileptic drugs response

**FIGURE 3:**
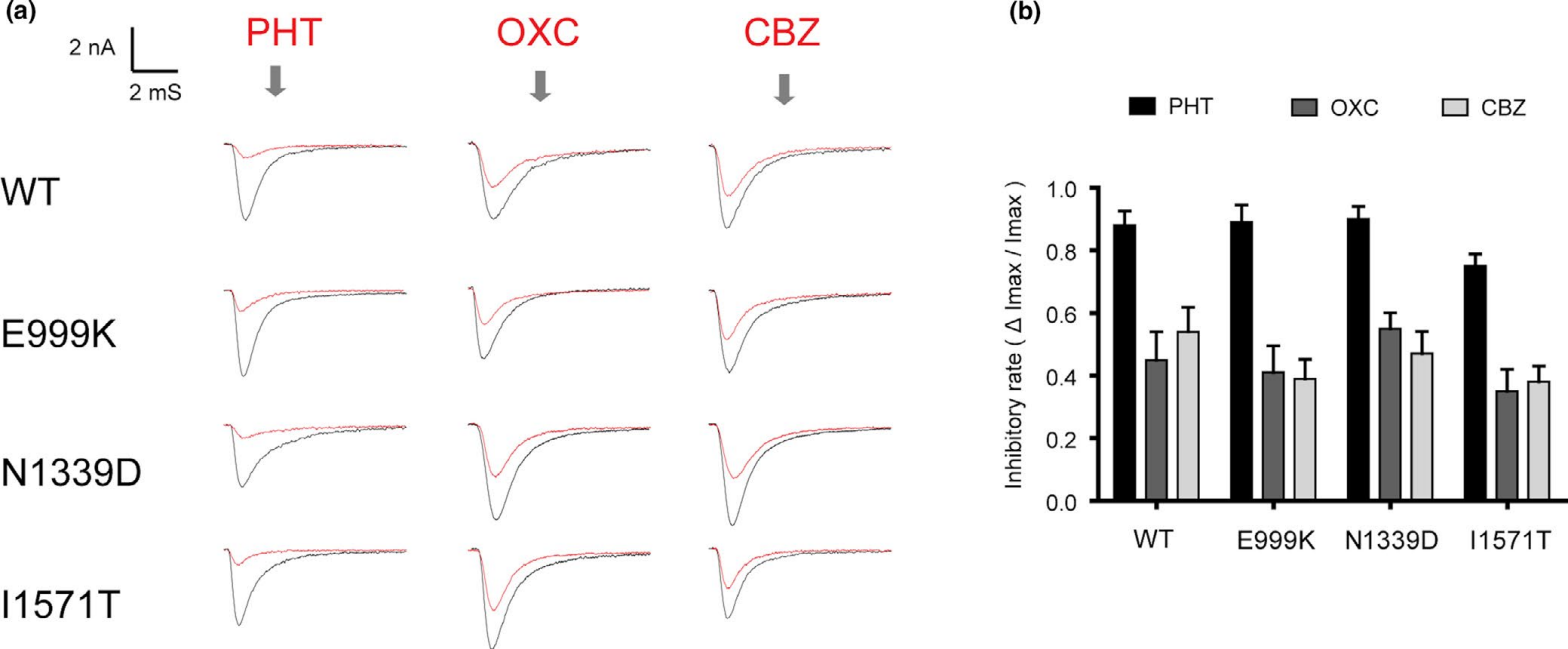
Antiepileptic drugs inhibit sodium currents of GOF mutations. (a) Representative currents recorded at −80 mV of WT and mutant Nav1.2 channels before and after application of 100 μM PHT, OXC, and CBZ. (b) Normalized inhibitory rate of sodium currents of antiepileptic drugs. Percentages of inhibition for WT and the mutations of Nav1.2 channels are evaluated by the peak current before and after application of drugs

Oxcarbazepine (OXC), carbamazepine (CBZ), and phenytoin (PHT), are commonly used to treat epileptic seizures. Their antiepileptic mechanisms are attributed to their inhibitory effects on sodium channels. The seizures in patients carrying the GOF variants (E999K, N1339D, and I1571T) were controlled partially or completely after administration of some of these drugs. The pharmacological electrophysiology results showed that the inhibitory effects of 100μM of both OXC and CBZ were similar. As the channels were exposed to OXC or CBZ, the peak amplitude of sodium currents was reduced by 35%~55%. Compared to these drugs, another classic antiepileptic drug (AED), PHT, was more effective in suppressing the function of Nav1.2. Almost 90% of the currents in the WT, E999K, and N1339D groups were blocked by 100 μM of PHT. In the participant with I1571P, PHT also inhibited over 70% of the peak current and was much more effective in doing so than OXC and CBZ (Figure 3b).

### 3.5 Previous reported variants

Similar to our study, 17 *SCN2A* variants detected in DEE had been functionally studied (Begemann et al., 2019; Berecki et al., 2018; Kamiya et al., 2004; Lauxmann et al., 2018; Liao et al., 2010; Lossin, Shi, Rogawski, & Hirose, 2012; Ogiwara et al., 2009; Papuc et al., 2018; Sawaishi, Yano, Enoki, & Takada, 2002; Wolff et al., 2017). Functional studies revealed variants with LOF effects in five patients and GOF in 10 patients. In two patients, the GOF effect was diminished by an additional LOF due to a hyperpolarizing shift of voltage‐dependent activation combined with either decreased amplitudes or an additional hyperpolarizing shift of the inactivation curve, which was defined as MIX. Phenotypes with LOF variants exhibited later age of seizure onset and extrapyramidal movement such as choreoathetosis. The details of the phenotype‐genotype‐electrophysiological features are shown in Table 2.

**TABLE 2:** Phenotype‒genotype and functional results from our and previous reported publication

Pub	Proteinchanges	Functionalchanged	S ageOnset age	Seizuretype	DD	Seizure improveWith SCBs	Other phenotypesphenotype
8	R102S	LOF	1 y 7 m	Atonic	Severe	—	Choreoathetosis
1	V423L	GOF	1 d	Tonic	—	No	Hypotonia
23	T733I	GOF	1 d	Myoclonic	Profound	No	
22	R853Q	LOF	>3 m	Spasms	Moderate	Worsen,PHT	Choreoathetosis
1	G899S	LOF	3 m	Hemiclonic	Moderate	Worsen, OXC	**—**
**/**	E999K	GOF	1 d	Tonic	Severe	Improved, PHT	**—**
9	E1211K	MIX	11 m	Spasms	Severe	No	Fever trigged
21	R1312T	LOF	Not clear	Not clear	—	—	Not clear
**/**	N1339D	GOF	2 m	Tonic	Severe	Improved, PHT	Brain atrophy
19	L1342P	GOF	Not clear	—	—	—	—
24	I1473M	GOF	1 m	Clonic	—	Improved, LID	**—**
**/**	I1571T	GOF	2 d	Tonic	Severe	Improved,OXC	—
1	F1597L	GOF	3 d	Tonic	Severe	Improved,PHT	Dysautonomia, irritability
**/**	P1658S	LOF	3m	Tonic	Moderate	No	Choreoathetosis
**/**	A1773T	LOF	11m	Spasms	—	Worsen.OXC	**—**
19,25	E1803G	GOF	3d	Myoclonic	Severe	Yes	**—**
22	R1882Q	GOF	1d	Focal	Severe	Improved,PHT	**—**

Note

Pub, means reference numbers of each variant;/, means variants from our study; Protein, protein change of each variant; Functional, functional change of each variant; LOF, loss of function; GOF, gain of function; MIX, mix of LOF and GOF; S age, seizure onset age; DD, degree of developmental delay; PHT, phenytoin; OXC, oxcarbazepine; Lid, Lidocaine;‐,means not mentioned from studies

## 4 DISCUSSION

Here, we studied the phenotypes, genotypes, and electrophysiological characteristics of five patients with five different de novo pathogenic variants of the *SCN2A*. This cohort embraces a multifarious phenotype of DEE, with the variants scattered across different domains with different electrophysiological functions, and it may therefore be a representative population. Seizure types vary widely between individuals, with patients exhibiting more than one type of seizure. Patients 1 and 3 had the earliest age of onset for tonic or tonic‐spasms seizure onset, exhibited burst suppression on the electroencephalogram (EEG), and were diagnosed with Ohtahara syndrome. In contrast, patients 4 and 5 presented with spasm and seizure onset after reaching 2 months of age, in accordance with the criteria for West syndrome. Developmental delays in these patients varied from moderate to profound global developmental delay.

All patients except the patient with the N1339D had normal cranial MRI images. N1339D was associated with progressive brain atrophy. Brain atrophy has been reported in four patients with the L1324P in *SCN2A* (Begemann et al., 2019; Dimassi et al., 2016; Matalon, Goldberg, Medne, & Marsh, 2014; Wolff et al., 2017) and two patients with I875T in *SCN3A* (Zaman et al., 2018), suggesting a potential variant‐specific effect on brain structural development.

Nav1.2(*SCN2A*) and other three paralogs of Nav1.1(*SCN1A*), Nav1.3(*SCN3A*), and Nav1.6(*SCN8A*) are the major sodium channels expressed throughout the human central nervous system. Variants in *SCN1A, SCN2A, SCN3A,* and *SCN8A* can therefore manifest similarly as infantile intractable epilepsy and severe ID (Johannesen et al., 2019; Lopez‐Santiago & Isom, 2019; Zaman et al., 2018). Furthermore, SCBs, especially PHT, produce a remarkably good response in patients with GOF variants in *SCN8A* and *SCN3A* but not in *SCN1A* (Boerma et al., 2016; Zaman et al., 2018)*.* In our study, there were three patients with GOF variants. The patient with p. I1571T successfully achieved seizure‐free status when treated with OXC, and the other two patients with p. N1339D and p.E999K showed little improvement following OXC treatment but showed remarkable improvement when treated with large doses of PHT. Katherine B. Howell et al. (2015) drew a similar conclusion and affirmed the effectiveness of PHT in a review of 46 cases of *SCN2A* encephalopathy. PHT is also an alternative approach used to manage difficult seizures in *SCN3A‐* and *SCN8A*‐related patients (Boerma et al., 2016; Zaman et al., 2018). In two patients in this study, the doses of PHT (12.5 mg/kg/d and 14 mg/kg/d) were higher than the loading doses (10 mg/kg/d) and were administered while monitoring PHT plasma concentrations. The nonlinear kinetics and risk of severe side effects of high PHT doses and the risk of neurological developmental injury associated with delaying dose adjustment are paradoxical (Brigo, 2012). Therefore, a “targeted loading approach” was developed for better personalized treatment (Welzel et al., 2019). We further tested the AEDs PHT, OXC, and CBZ in heterologous Nav1.2 variants with p. N1339D or p. E999K, and the results showed that almost 90% of the currents in the WT, E999K, and N1339D groups were blocked by 100 μM of PHT. We concluded that PHT produced the largest decrease, in accordance with clinical manifestations.

Patient 2 in our study had a previously unpublished de novo variant (c.4972C>T, p. P1658S). The P1658S mutation of Nav1.2 causes a complete LOF that produced no detectable channel currents. The phenotype presented as tonic seizures. At 3 months of age, the patient presented the motions of choreoathetosis with paroxysmal intractable vomiting lasting for 2 days. The patient was treated with OXC for nearly 3 years until we acquired the LOF results in a functional study, and then decided to avoid using SCBs. In accordance with previously published investigations, SCBs are rarely effective in epilepsies with later onset (≥3 months), and account for approximately 20‒40% of cases (Wolff et al., 2017). More precise pediatric epilepsy diagnoses are now possible by WES (Sharma, Hussain and Greenwood, 2019). These precision diagnoses support the development of personalized therapies by providing insights into genotype‒phenotype relationships; for example, we would not use SCBs in *SCN1A*‐related epilepsy (Brunklaus, Ellis, Reavey, Forbes, & Zuberi, 2012). In practice, a *SCN1A* GOF variant has been acknowledged to lead to early infantile encephalopathy based on whole‐cell patch clamp findings, and in such cases, SCBs could show unexpected efficacy (Berecki et al., 2019). Therefore, future treatments should be based on an advanced understanding of both GOF and LOF phenotypes.

Patient 5 (A1773T) had another pathogenic variant that showed LOF characteristics. The OXC exacerbated her seizures, and the patient was found to exhibit involuntary dancing movements. However, whole‐cell voltage‐clamp analysis revealed a MIX function: hyperpolarized activation and inactivation with slower recovery from inactivation. While the fast‐inactivation time constant τ1 of A1773T was significantly larger than that found in WT, in combination with clinical features, we postulated that this variant produced a LOF effect. R1312T and E1211K were found to result in the same phenomenon (Lossin et al., 2012; Ogiwara et al., 2009). Another case of a patient carrying a de novo A1773T variant was previously been reported (Wolff et al., 2017). This patient showed seizure onset at 3 years and 6 months of age and was categorized as encephalopathy with late onset epilepsy. LEV administration led to seizure‐free status at 4 years of age. The difference between the published patient and our patient was the neurological features. The published report patient with A1773V had ASD, while our patient showed choreoathetosis.

Recently, an increasing number of homologous structures of Nav channels have been identified. By studying these structures, we can gain a better understanding of how different variants affect the channel's function. Access to homologous structures allowed the generation of a complete three‐dimensional model for *SCN2A* (Yan et al., 2017, Shen et al., 2017, Pan et al., 2018, Pan et al., 2019) and a comprehensive evaluation of the effects of pathogenic variants. Previous studies have demonstrated that the hydrophobic cluster Ile/Phe/Met (IFM) motif in the III‐IV linker is essential for the fast inactivation of Nav channels (Vassilev, Scheuer, & Catterall, 1989; West et al., 1992). Some residues on the S4–S5 linker and S6 segments of domains III and IV are also involved in this process (Goldin, 2003; Ulbricht, 2005). As shown in Figure 2, the p. P1658S variant in this study was located in the S4–S5 linker of IV, while p. A1773T variant was located in the S6 segments of IV. Both these sites are close to the IFM motif, suggesting a potential interaction with it. Variants that lead to amino acid changes at these two mutant sites could interfere with the interactions between them or alter the characterization of inactivation. Next, with respect to the P1658 site, A1659 and N1662 are in direct contact with the IFM motif. The substitution of P1658 by Ser could change the conformation of the α‐helix, causing many interactions with the IFM motif to be disturbed, leading to complete LOF.

**FIGURE 4:**
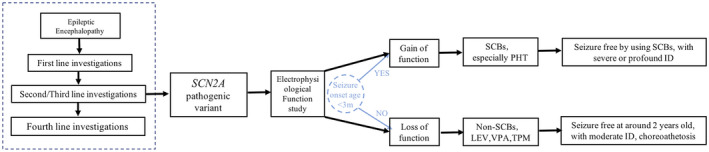
The protocol of SCN2A‐related developmental epileptic encephalopathy. First‐line investigation: plasma amino acids, homocysteine, urine organic acids, acylcarnitine profile, total and free carnitine, urine GAA and creatine, urine alpha‐AASA, aCGH, and brain MRI. Second/Third‐line investigations: targeted next generation sequencing panels of developmental epileptic encephalopathy genes or whole‐exome sequencing. Fourth‐line investigations: muscle biopsy, skin biopsy, and CSF metabolic investigations. Electrophysiological function study is the golden standard of judging GOF of LOF variants. Seizure onset age could be considered as a preliminarily judging method, which should be verified by the whole‐cell patch‐clamp

To date, nearly 700 known *SCN2A* cases have been identified, but only about 13% of the related pathogenic variants have been functionally assessed. Those that have been assessed included 12 *SCN2A* variants detected in DEE. Patients with GOF variants are nearly two times more common as compared to LOF variants. The genotype‐phenotype relationships observed in epilepsy might be more complex when both GOF and LOF changes are observed (Oyrer et al., 2018). GOF variants produced distinguishing phenotypic features, including seizure onset before 3 months of age, with 77% occurring before 3 days after birth, in combination with severe to profound mental retardation. Patients with LOF variants also exhibit their own characteristics: seizure onset after 3 months old, with 80% occurring between 3 and 12 months old, in combination with moderate mental retardation and characteristic choreoathetoid movements. Previously, only limited *SCN2A*‐specific data were available for patients with seizures starting between 3 and 12 months of age. However, in our study, we included two patients in this study and those from two published cases, all of whom had seizure onset between 3 and 12 months of age and showed the features as LOF variants. Using the phenotypic classification described above, based on the seizure onset age, cognitive outcomes, and movement disorders observed in these patients, we first evaluated the function of unpublished de novo pathogenic variants to assist us in selecting precision treatment and determining the implications of our findings on prognosis. However, because genotype‐phenotype relationships are so complex, whole‐cell voltage clamp is the gold standard and next precise step. We propose that the whole‐cell voltage‐clamp approach is as important as WES in patients with pathogenic variants in genes related to ion channels. In conclusion, we formulated a system for a diagnosis and treatment protocol for *SCN2A*‐related DEE (Figure 4).

To date, neuronal hyperexcitability because of GOF variants in *SCN2A* most likely contribute to infantile epilepsy, but how can our study explain that LOF variants (haploinsufficiency) in *SCN2A* diminishing or eliminating channel function also lead to DEE with relatively milder phenotype by our study? The mechanisms underlying the association between *SCN2A* LOF and DEE remain largely unknown. However, our study showed that there is still a distinction between GOF and LOF of DEE patients, which we can be made by the early identification from phenotypes and the next step is to figure out the funotypes of each variant.

## 5 CONCLUSION

In conclusion, we enrolled five patients and reviewed 12 functionally studied cases with *SCN2A*‐related DEE and obtained a primary diagnosis and treatment protocol. For patients with a seizure onset earlier than 3 months of age and more severe developmental delay, SCBs, especially PHT, should be optimized. However, in patients with seizure onset after reaching 3 months of age with choreoathetosis, SCBs should be avoided, and the patient may exhibit relatively moderate developmental delay.

## CONFLICTS OF INTEREST

The authors declare that they have no conflict of interest.

## AUTHOR CONTRIBUTIONS

JH.F, YT.L, BJ.Z, XX.X, and XQ.C diagnosed patients and collected data, SY.T and J.Y performed the electrophysiological studies and analyses, P.M., SY.T, J.Y, JH.F, and YZ.L were responsible for the conception and design of the study, P.M and SY.T contributed to drafting the text and preparing the figures.

## Supporting information

Table S1

## References

[mgg31250-bib-0001] Begemann, A. , Acuña, M. A. , Zweier, M. , Vincent, M. , Steindl, K. , Bachmann‐Gagescu, R. , … Rauch, A. (2019). Further corroboration of distinct functional features in SCN2A variants causing intellectual disability or epileptic phenotypes. Molecular Medicine, 25(1), 10.1186/s10020-019-0073-6 PMC6391808 30813884

[mgg31250-bib-0002] Ben‐Shalom, R. , Keeshen, C. M. , Berrios, K. N. , An, J. Y. , Sanders, S. J. , & Bender, K. J. (2017). Opposing effects on Na V 1.2 function underlie differences between SCN2A variants observed in individuals with autism spectrum disorder or infantile seizures. Biological Psychiatry, 82, 224–232. 10.1016/j.biopsych.2017.01.009 28256214 PMC5796785

[mgg31250-bib-0003] Berecki, G. , Bryson, A. , Terhag, J. , Maljevic, S. , Gazina, E. V. , Hill, S. L. , & Petrou, S. (2019). SCN1A gain of function in early infantile encephalopathy. Annals of Neurology, 85, 514–525. 30779207 10.1002/ana.25438

[mgg31250-bib-0004] Berecki, G. , Howell, K. , Deerasooriya, Y. , Cilio, M. , Oliva, M. , Kaplan, D. , … Petrou, S. (2018). Dynamic action potential clamp predicts functional separation in mild familial and severe de novo forms of SCN2A epilepsy. Proceedings of the National Academy of Sciences of the United States of America, 115, E5516–E5525. 10.1073/pnas.1800077115 29844171 PMC6004444

[mgg31250-bib-0005] Boerma, R. S. , Braun, K. P. , van den Broek, M. P. , van Berkestijn, F. M. , Swinkels, M. E. , Hagebeuk, E. O. , … Koeleman, B. P. (2016). Remarkable phenytoin sensitivity in 4 children with SCN8A‐related Epilepsy: A molecular neuropharmacological approach. Neurotherapeutics: The Journal of the American Society for Experimental NeuroTherapeutics, 13, 192–197. 10.1007/s13311-015-0372-8 26252990 PMC4720675

[mgg31250-bib-0006] Brigo, F. (2012). Phenytoin is dead, long live phenytoin? Epilepsy and Behavior, 24, 152. 22481039 10.1016/j.yebeh.2012.02.018

[mgg31250-bib-0007] Brunklaus, A. , Ellis, R. , Reavey, E. , Forbes, G. H. , & Zuberi, S. M. (2012). Prognostic, clinical and demographic features in SCN1A mutation‐positive Dravet syndrome. Brain, 135, 2329–2336. 10.1093/brain/aws151 22719002

[mgg31250-bib-0008] Dimassi, S. , Labalme, A. , Ville, D. , Calender, A. , Mignot, C. , Boutry‐Kryza, N. , … Lesca, G. (2016). Whole‐exome sequencing improves the diagnosis yield in sporadic infantile spasm syndrome. Clinical Genetics, 89, 198–204. 10.1111/cge.12636 26138355

[mgg31250-bib-0009] Gazina, E. V. , Leaw, B. T. , Richards, K. L. , Wimmer, V. C. , Kim, T. H. , Aumann, T. D. , … Petrou, S. (2015). 'Neonatal' Nav1.2 reduces neuronal excitability and affects seizure susceptibility and behaviour. Human Molecular Genetics, 24, 1457–1468. 25378553 10.1093/hmg/ddu562

[mgg31250-bib-0010] Goldin, A. L. (2003). Mechanisms of sodium channel inactivation. Current Opinion in Neurobiology, 13, 284–290. 10.1016/S0959-4388(03)00065-5 12850212

[mgg31250-bib-0011] Herzog, R. I. , Cummins, T. R. , Ghassemi, F. , Dib‐Hajj, S. D. , & Waxman, S. G. (2003). Distinct repriming and closed‐state inactivation kinetics of Nav1.6 and Nav1.7 sodium channels in mouse spinal sensory neurons. Journal of Physiology, 551, 741–750. 10.1113/jphysiol.2003.047357 12843211 PMC2343279

[mgg31250-bib-0012] Howell, K. , McMahon, J. , Carvill, G. , Tambunan, D. , Mackay, M. , Rodriguez‐Casero, V. , … Scheffer, I. (2015). SCN2A encephalopathy: A major cause of epilepsy of infancy with migrating focal seizures. Neurology, 86, 958–966. 10.1212/WNL.0000000000001926 PMC4567464 26291284

[mgg31250-bib-0013] Johannesen, K. M. , Gardella, E. , Encinas, A. C. , Lehesjoki, A. E. , Linnankivi, T. , Petersen, M. B. , … Moller, R. S. (2019). The spectrum of intermediate SCN8A‐related epilepsy. Epilepsia, 60, 830–844. 30968951 10.1111/epi.14705

[mgg31250-bib-0014] Kamiya, K. , Kaneda, M. , Sugawara, T. , Mazaki, E. , Okamura, N. , Montal, M. , … Yamakawa, K. (2004). A nonsense mutation of the sodium channel gene SCN2A in a patient with intractable epilepsy and mental decline. Journal of Neuroscience, 24, 2690–2698. 10.1523/JNEUROSCI.3089-03.2004 15028761 PMC6729532

[mgg31250-bib-0015] Laezza, F. , Lampert, A. , Kozel, M. A. , Gerber, B. R. , Rush, A. M. , Nerbonne, J. M. , … Ornitz, D. M. (2009). FGF14 N‐terminal splice variants differentially modulate Nav1.2 and Nav1.6‐encoded sodium channels. Molecular and Cellular Neurosciences, 42, 90–101. 10.1016/j.mcn.2009.05.007 19465131 PMC2832592

[mgg31250-bib-0016] Lauxmann, S. , Boutry‐Kryza, N. , Rivier, C. , Mueller, S. , Hedrich, U. , Maljevic, S. , … Lesca, G. (2013). An SCN2A mutation in a family with infantile seizures from Madagascar reveals an increased subthreshold Na(+) current. Epilepsia, 54, e117–121. 23758435 10.1111/epi.12241

[mgg31250-bib-0017] Lauxmann, S. , Verbeek, N. E. , Liu, Y. , Zaichuk, M. , Muller, S. , Lemke, J. R. , … Hedrich, U. B. S. (2018). Relationship of electrophysiological dysfunction and clinical severity in SCN2A‐related epilepsies. Human Mutation, 39, 1942–1956. 10.1002/humu.23619 30144217

[mgg31250-bib-0018] Liao, Y. , Deprez, L. , Maljevic, S. , Pitsch, J. , Claes, L. , Hristova, D. , … Lerche, H. (2010). Molecular correlates of age‐dependent seizures in an inherited neonatal‐infantile epilepsy. Brain, 133, 1403–1414. 10.1093/brain/awq057 20371507

[mgg31250-bib-0019] Lopez‐Santiago, L. , & Isom, L. L. (2019). Dravet syndrome: A developmental and epileptic encephalopathy. Epilepsy Currents, 19, 51–53. 10.1177/1535759718822038 30838929 PMC6610375

[mgg31250-bib-0020] Lossin, C. , Shi, X. , Rogawski, M. A. , & Hirose, S. (2012). Compromised function in the Na(v)1.2 Dravet syndrome mutation R1312T. Neurobiology of Diseases, 47, 378–384. 10.1016/j.nbd.2012.05.017 22677033

[mgg31250-bib-0021] Matalon, D. , Goldberg, E. , Medne, L. , & Marsh, E. D. (2014). Confirming an expanded spectrum of SCN2A mutations: A case series. Epileptic Disord, 16, 13–18. 10.1684/epd.2014.0641 24659627

[mgg31250-bib-0022] Miao, P. , Feng, J. , Guo, Y. , Wang, J. , Xu, X. , Wang, Y. , … Cheng, H. (2018). Genotype and phenotype analysis using an epilepsy‐associated gene panel in Chinese pediatric epilepsy patients. Clinical Genetics, 94, 512–520. 10.1111/cge.13441 30182498

[mgg31250-bib-0023] Ogiwara, I. , Ito, K. , Sawaishi, Y. , Osaka, H. , Mazaki, E. , Inoue, I. , … Yamakawa, K. (2009). De novo mutations of voltage‐gated sodium channel alphaII gene SCN2A in intractable epilepsies. Neurology, 73, 1046–1053. 19786696 10.1212/WNL.0b013e3181b9cebc PMC2754324

[mgg31250-bib-0024] Oyrer, J. , Maljevic, S. , Scheffer, I. E. , Berkovic, S. F. , Petrou, S. , & Reid, C. A. (2018). Ion Channels in Genetic Epilepsy: From Genes and Mechanisms to Disease‐Targeted Therapies. Pharmacological Reviews, 70, 142–173. 10.1124/pr.117.014456 29263209 PMC5738717

[mgg31250-bib-0025] Pan, X. , Li, Z. , Huang, X. , Huang, G. , Gao, S. , Shen, H. , … Yan, N. (2019). Molecular basis for pore blockade of human Na+ channel Nav1.2 by the μ‐conotoxin KIIIA. Science, 363, 1309–1313. 30765605 10.1126/science.aaw2999

[mgg31250-bib-0026] Pan, X. , Li, Z. , Zhou, Q. , Shen, H. , Wu, K. , Huang, X. , … Yan, N. (2018). Structure of the human voltage‐gated sodium channel Nav1.4 in complex with β1. Science, 362, eaau2486. 30190309 10.1126/science.aau2486

[mgg31250-bib-0027] Papuc, S. M. , Abela, L. , Steindl, K. , Begemann, A. , Simmons, T. L. , Schmitt, B. , … Rauch, A. (2018). The role of recessive inheritance in early‐onset epileptic encephalopathies: A combined whole‐exome sequencing and copy number study. European Journal of Human Genetics, 27, 408–421. 10.1038/s41431-018-0299-8 30552426 PMC6460568

[mgg31250-bib-0028] Rush, A. M. , Dib‐Hajj, S. D. , & Waxman, S. G. (2005). Electrophysiological properties of two axonal sodium channels, Nav1.2 and Nav1.6, expressed in mouse spinal sensory neurones. Journal of Physiology, 564, 803–815. 15760941 10.1113/jphysiol.2005.083089 PMC1464456

[mgg31250-bib-0029] Sakmann, B. , & Neher, E. (1984). Patch clamp techniques for studying ionic channels in excitable membranes. Annual Review of Physiology, 46, 455–472. 10.1146/annurev.ph.46.030184.002323 6143532

[mgg31250-bib-0030] Sanders, S. J. , Campbell, A. J. , Cottrell, J. R. , Moller, R. S. , Wagner, F. F. , Auldridge, A. L. , … Bender, K. J. (2018). Progress in understanding and treating SCN2A‐mediated disorders. Trends in Neurosciences, 41, 442–456. 10.1016/j.tins.2018.03.011 29691040 PMC6015533

[mgg31250-bib-0031] Sawaishi, Y. , Yano, T. , Enoki, M. , & Takada, G. (2002). Lidocainedependent early infantile status epilepticus with highly suppressed EEG. Epilepsia, 43, 201–204. 10.1046/j.1528-1157.2002.25301.x 11903470

[mgg31250-bib-0032] Schwarz, N. , Hahn, A. , Bast, T. , Muller, S. , Loffler, H. , Maljevic, S. , … Hedrich, U. B. S. (2016). Mutations in the sodium channel gene SCN2A cause neonatal epilepsy with late‐onset episodic ataxia. Journal of Neurology, 263, 334–343. 10.1007/s00415-015-7984-0 26645390

[mgg31250-bib-0033] Sharma, P. , Hussain, A. , & Greenwood, R. (2019). Precision in pediatric epilepsy. F1000Res, , 8, 163 10.12688/f1000research.16494.1 PMC6367658 30800292

[mgg31250-bib-0034] Shen, Huaizong , Zhou, Qiang , Pan, Xiaojing , Li, Zhangqiang , Wu, Jianping , & Yan, Nieng (2017). Structure of a eukaryotic voltage‐gated sodium channel at near‐atomic resolution. Science, 355(6328), eaal4326 10.1126/science.aal4326 28183995

[mgg31250-bib-0035] Ulbricht, W. (2005). Sodium channel inactivation: Molecular determinants and modulation. Physiological Reviews, 85, 1271–1301. 10.1152/physrev.00024.2004 16183913

[mgg31250-bib-0036] Vassilev, P. , Scheuer, T. , & Catterall, W. (1989). Inhibition of inactivation of single sodium channels by a site‐directed antibody. Proceedings of the National Academy of Sciences of the United States of America, 86, 8147–8151. 10.1073/pnas.86.20.8147 2554301 PMC298232

[mgg31250-bib-0037] Welzel, T. , Ziesenitz, V. C. , Waldvogel, S. , Scheidegger, S. , Weber, P. , van den Anker, J. N. , & Gotta, V. (2019). Use of a personalized phenytoin dosing approach to manage difficult to control seizures in an infant with a SCN2A mutation. European Journal of Clinical Pharmacology, 75, 737–739. 10.1007/s00228-019-02629-w 30643928

[mgg31250-bib-0038] West, J. W. , Patton, D. E. , Scheuer, T. , Wang, Y. , Goldin, A. L. , & Catterall, W. A. (1992). A cluster of hydrophobic amino acid residues required for fast Na(+)‐channel inactivation. Proceedings of the National Academy of Sciences of the United States of America, 89, 10910–10914. 10.1073/pnas.89.22.10910 1332060 PMC50452

[mgg31250-bib-0039] Wolff, M. , Johannesen, K. M. , Hedrich, U. B. S. , Masnada, S. , Rubboli, G. , Gardella, E. , … Møller, R. S. (2017). Genetic and phenotypic heterogeneity suggest therapeutic implications in SCN2A‐related disorders. Brain, 140, 1316–1336. 10.1093/brain/awx054 28379373

[mgg31250-bib-0040] Yan, Z. , Zhou, Q. , Wang, L. , Wu, J. , Zhao, Y. , Huang, G. , … Yan, N. (2017). Structure of the Nav1.4‐β1 Complex from Electric Eel. Cell, 170, 470–482.e11. 28735751 10.1016/j.cell.2017.06.039

[mgg31250-bib-0041] Zaman, T. , Helbig, I. , Bozovic, I. B. , DeBrosse, S. D. , Bergqvist, A. C. , Wallis, K. , … Goldberg, E. M. (2018). Mutations in SCN3A cause early infantile epileptic encephalopathy. Annals of Neurology, 83, 703–717. 29466837 10.1002/ana.25188 PMC5912987

