# Supplementary material for: Electrophysiological features: The next precise step for SCN2A developmental epileptic encephalopathy
Source: Mol Genet Genomic Med. 2020 May 13;8(7):e1250. doi: 10.1002/mgg3.1250 (PMC7336724; doi:10.1002/mgg3.1250)
Supplement: Supplementary file 1 — Table S1 [file MGG3-8-e1250-s001.docx]

**Table 1 Figures of CADD and Polyphen and Gnomad database of these five variants**

| Protein | **p.I1571T** | **p.P1658S** | **p.E999K** | **p.N1339D** | **p.A1773T** |
| --- | --- | --- | --- | --- | --- |
| CADD_raw | 4.132 | 5.259 | 5.865 | 5.862 | 5.971 |
| CADD_raw_rankscore | 0.554 | 0.711 | 0.804 | 0.803 | 0.82 |
| CADD_phred | 23.8 | 25.7 | 27.3 | 27.3 | 27.7 |
| Polyphen2_HDIV_score | 0.998 | 1 | 0.971 | 0.997 | 1 |
| Poly2_HDIV_rankscore | 0.715 | 0.899 | 0.556 | 0.715 | 0.899 |
| Polyphen2_HDIV_pred | D | D | D | D | D |
| Poly2_HVAR_rankscore | 0.875 | 0.971 | 0.589 | 0.875 | 0.916 |
| Polyphen2_HVAR_pred | D | D | P | D | D |
| Gnomad | - | - | - | - | - |
| ACMG scoring | PS2+PM2+PP3 | PS2+PM2+PP3 | PS1+PS2+PM1+PM2+PP3 | PS2+PM2+PP3 | PS1+PS2+PM2+PP3 |
